# Receptor-like kinase SlRLK-like positively regulates sugar accumulation and fruit ripening in tomato

**DOI:** 10.3389/fpls.2025.1649082

**Published:** 2025-08-20

**Authors:** Jiaqi Sun, Xinsheng Zhang, Miaofei Yang, Xin Liu, Jing Jiang

**Affiliations:** ^1^ College of Horticulture, Shenyang Agricultural University, Shenyang, China; ^2^ College of Agriculture, Liaodong University, Dandong, China; ^3^ College of Horticulture, Jilin Agricultural University, Changchun, China

**Keywords:** SlRLK-like, *Cr*RLK1Ls, SlSWEETs, Suc, ethylene, lycopene, tomato, fruit ripening

## Abstract

**Introduction:**

The ripening process of tomato fruits involves many complex changes. The elucidation of the ripening pathways contributes to the reduction of post-harvest losses and improvement of fruit quality. However, much is unknown about how tomato plants precisely synchronize metabolic regulation and fruit maturation.

**Methods:**

VIGS, MY2H, BiFC, GUS fused protein activity assays, western-blot, co-expressing in *Nicotiana benthamiana* leaves and assays of esculin fluorescence in yeast cells, etc.

**Results:**

In this study, SlRLK-like, a member of the *Catharanthus roseus* receptor-like kinase 1-like (*Cr*RLK1L) in tomato, was found to be involved in the regulation of sugar transport, lycopene content, and synthesis of ethylene. The overexpression (OE) or virus-induced gene silencing (VIGS) of *SlRLK-like* increases or decreases sugar accumulation in tomato fruits, respectively. Meanwhile, overexpressing *SlRLK-like* causes the acceleration of the ripening process of tomato fruit, which also results in the upregulation of ethylene production and lycopene content. *SlRLK-like* can interact with SlSWEETs (SlSWEET7a and SlSWEET14) and further alter their sucrose transport ability to modulate sugar accumulation. Furthermore, during tomato fruit ripening, SlRLK-like proteins can also interact with ethylene and lycopene biosynthesis-related proteins (SlACS2, SlSAMS4, and SlPSY1) by controlling their gene expression level and protein abundance to promote the process of lycopene and ethylene production. More importantly, sugar accumulation in fruit can enhance ethylene production, which can also speed up the tomato fruit ripening process.

**Discussion:**

As a result, SlRLK-like acts as a positive regulator of tomato ripening. The study provides novel insights into the molecular regulatory networks of tomato fruit quality and ripening, which can be applied to improve tomato cultivation.

## 1 Introduction

*Catharanthus roseus* receptor-like kinase 1-like protein family (*Cr*RLK1Ls), also designated as M/MLD-RLKs, have two malectin-like domains between the transmembrane motif (TM) and N-terminal SP (signal peptide) (Schulze-Muth et al., 1996; Yang et al., 2021). Malectin binds to carbohydrates and is a candidate for protein N-glycosylation, suggesting that M/MLD-RLKs recognize carbohydrate-rich structures (Schallus et al., 2008; Boisson-Dernier et al., 2011; Galli et al., 2011). M/MLD-RLKs are divided into THESEUS1 (THE) and FERONIA (FER) sub-clades (Yang et al., 2021). There has been significant progress in understanding how M/MLD-RLKs regulate plant cell elongation (Gonneau et al., 2018; Hansen et al., 2019; Zhu et al., 2020), pollen tube development (Zhu et al., 2018; Duan et al., 2020; Galindo-Trigo et al., 2020; Pu et al., 2017; Zhang et al., 2021a), and responses to environmental stimuli (Mang et al., 2017; Yin et al., 2018; Kim et al., 2021). A few FER subfamily members have been reported to play roles in fruit quality. FaMRLK47—a FERL (FER-like RLK)—can regulate fruit ripening and quality by interacting with FaABI1 (Jia et al., 2017a). Additionally, MdFERL1 and MdFERL6 regulate the production of ethylene by interacting with MdSAMS. OE-*MdFERL6* delays apple ripening while suppressing ethylene production (Jia et al., 2017b). In tomatoes, *SlFERL* significantly alters tomato fruit ripening (Ji et al., 2020). In contrast to the FER sub-clade, advances in understanding the THE sub-clade members in fruit quality remain limited. Recently, SlRLK-like, a member of the THE subfamily, has been shown to negatively regulate ethylene biosynthesis when induced by EIX (Sussholz et al., 2020). Nevertheless, the functions of the THE subfamily members in tomato have been less studied. We wonder about the further roles that the THE subfamily members play in tomato fruit quality traits and in regulating ripening.

One of the most significant horticultural crops in the world and a significant source of nutrients is tomatoes (*Solanum lycopersicum*) (Centeno et al., 2011). Improving tomato quality requires the storage and transfer of sugar (Shammai et al., 2018), with sugar transporters playing indispensable roles in sugar accumulation (Ruan, 2014; Li et al., 2017; Zhang et al., 2018). Sugars will eventually be exported transporter (SWEET) proteins play a role in sugar transport (Eom et al., 2015). Recently, several SlSWEET family members have been shown to play roles in sugar accumulation during tomato development. More specifically, the SlSWEET15 facilitator contributes to the expansion stage of fruit development by unloading Suc from the phloem (Ko et al., 2021). During tomato ripening, SlSWEET1a regulates Glu content and the Fru : Glu ratio (Shammai et al., 2018). Meanwhile, SlSWEET14 interacts with SlSWEET7a to regulate sugar storage and transport in tomatoes (Zhang et al., 2021b). SlSWEET10a, functioning as another SlSWEET14 interactor, has been reported to negatively regulate sucrose transport in tomato fruit (Zhang et al., 2024). Additionally, *SlSWEET12c* is highly expressed during the tomato red ripening (RR) stage and participates in Suc effusion (Sun et al., 2022). It has been found that some transcription factors modulate the sugar distribution by regulating the expression of the SWEET gene in cotton, *Arabidopsis*, and pear (Sun et al., 2019; Huang et al., 2020; Li et al., 2020). However, it is currently not known whether SWEET family proteins play a role in regulating translation.

Sugar composition and quantity are important factors in energy metabolism and fruit flavor. Additionally, sugars also have a signaling function similar to that of hormones (Durán-Soria et al., 2020). In both climacteric and non-climacteric fruit, ethylene is believed to be necessary for the Suc-induced regulation of carotenoid accumulation (Iglesias et al., 2001; Télef et al., 2006; Lu and Zhu, 2022). Exogenous Suc treatment accelerates the ripening of post-harvest tomato fruits by modulating their metabolism, ethylene biosynthesis, and signal transduction (Li et al., 2016). Meanwhile, ACC oxidase activity is typically reduced by 60%–70% in Glu-treated tomato fruits (Hong et al., 2004). During strawberry fruit ripening, exogenous Suc exerts the most significant effect on ripening, followed by Glc (Jia et al., 2011). SlVIF, encoding a vacuolar invertase inhibitor, physically interacts with SlVI to control Suc metabolism and the biosynthesis of ethylene (Qin et al., 2016). Altogether, sugars play a role in regulating the biosynthesis of ethylene during fruit ripening.

Previously, our team identified that SlSWEET14 contributes to the storage and transport of sugar in tomato fruits (Zhang et al., 2021b). In this current study, we screened for proteins that interact with the SlSWEET14 protein. We described here the subsequent characterization of an associated M/MLD-RLK, SlRLK-like. We speculated that SlRLK-like plays a role in tomato fruit development by interacting with SlSWEETs. The RT-qPCR and GUS analysis of SlRLK-like showed that in tomato fruit, it had a high expression level. The overexpression (OE) and virus-induced gene silencing (VIGS) of *SlRLK-like* caused alterations in sugar accumulation and the ripening process in tomato fruit. Further study showed that SlRLK-like interacted with SlSWEET7a and SlSWEET14 to regulate the sugar accumulation. Also, SlRLK-like modulated the ethylene biosynthesis pathway and lycopene accumulation. We identified the targets for enhancing tomato fruit quality and ripening characteristics and manipulated them in this work.

## 2 Materials and methods

### 2.1 Plant materials and growth conditions

Tomato (*S. lycopersicum*) wild type ‘Micro-Tom’ (MT) was selected as background and control lines due to its short growth cycle.

The growth conditions of MT plants were as follows: 25–30°C, 16-h light and 8-h dark illumination, and 70%–75% humidity. The source leaves were collected at 30 days after germination, and fruits, including IMG, MG, BR, and RR, which were respectively referred to as immature green, mature green, breaker, and red ripening, were collected at 30, 35, 40, and 45 days post-anthesis (dpa), respectively.

### 2.2 Total RNA isolation and real-time fluorescence quantitative PCR analysis

Extraction of the total RNA of various tomato tissues was performed using the TRIzol kit (Tiangen, Beijing, China) following the instructions of the manufacturer. For the expression pattern analysis, the fruit samples were collected without seeds and epicarp. The extracted total RNA was reverse-transcribed. Then, the PrimeScript RT Master Mix kit (Takara, Dalian, China) was used to synthesize the cDNA according to the instructions. A RT-qPCR analysis of the synthesized cDNA was performed with the Bio-Rad CFX96 Real-Time PCR System instrument using the SuperReal PreMix Plus (SYBR Green) kit (Tiangen, Beijing, China). The quantitative internal control gene was *Actin* (the ACTB encoding gene); the 2^−ΔΔCt^ calculation method was used to determine the fold-change (Zhang et al., 2021b). The number of RNA isolation and RT-qPCR samples was ≧3. The primers are listed in **Supplementary Table S1**.

### 2.3 *SlRLK-like*-GUS fused protein activity assays

The promoter of *SlRLK-like* (−1,500 bp) was cloned into the pBGWES7.0 vector with a GUS target using the primer in **Supplementary Table S1**. The *SlRLK-like-GUS* vector was introduced into GV3101 (an *Agrobacterium* strain), and MT was stained using a GUS kit (Tiangen, Beijing, China) to stain IMG, MG, BR, and RR of T_0_ generation fruits and then observed under a Nikon SMZ800 stereo microscope.

### 2.4 Generation of *SlRLK-like* overexpressing plants

The coding region of *SlRLK-like* (without stop codons), alongside the *Sac*I and *Xma*I cleavage sites, was cloned into pCAMBIA3301-Luc (containing the CaMV35S promoter). The resulting construct was transformed into GV3101 and further into MT via the leaf disc method using 60 µg/mL phosphinothricin (PPT) to select positive plants. Non-segregating homozygous lines were chosen from three lines of the T_1_ generation. T_4_ generation lines were used for our further analysis.

### 2.5 Virus-induced gene silencing

*SlRLK-like*-, *SlACS2*-, *SlPSY1*-, and *SlSAMS4*-specific sequences (300 bp each), which were obtained using the VIGS Tool (Sol Genomics Network; https://solgenomics.net/), were then introduced into the pTRV2 empty vector together with the *Kpn*I site using primers in **Supplementary Table S1**. Both fused vectors and the pTRV1 empty vector were transformed into GV3101. The strains that carried pTRV1 and pTRV2 or the fused vectors were mixed in a 1:1 ratio and then injected into the inflorescence peduncles attached to the MT fruit, as previously described (Chen et al., 2010). RT-qPCR was also performed to determine the virus accumulation and the efficiency of silencing specific genes in transgenic tomato fruits.

### 2.6 Co-expressing in *Nicotiana benthamiana* leaves

The coding sequences (CDSs) of SlSWEET7a, SlSWEET14, SlRLK-like, SlACS2, SlPSY1, and SlSAMS4 were cloned into pCAMBIA1300-mCherry vector. SlSWEET12c was used as a plasma membrane marker. The fused vectors were transformed into the GV3101 strain and then injected into the leaves of *N. benthamiana* in pairs. Forty-eight hours after injection, Leica SP8 (a confocal laser-scanning microscope; Wetzlar, Germany) was used to observe the fluorescence signal. The excitation wavelength was either 488 or 561 nm. The emission wavelength was either 500–572 or 605–635 nm.

### 2.7 Western blotting

Co-expressed *N. benthamiana* leaves with a mass of 0.2 g (described in Section 2.6) were collected, and protein was extracted using the Solarbio plant protein extraction kit. Then, the Solarbio BCA protein assay kit was used to assay the protein concentration. Sodium Dodecyl Sulfate PolyAcrylamide Gel Electrophoresis (SDS–PAGE) was used to analyze protein samples, and blotting was conducted using anti-mCherry and anti-β-actin (internal control), both from ABclonal (Wuhan, China).

### 2.8 Sugar content measurement

Fruit samples with a mass of 0.5 g, including MG, BR, and RR fruits from wild-type (WT) and SlRLK-like transgenic plants, were collected; 80% (v/v) ethanol and high-performance liquid chromatography were used to extract and analyze Suc, Glu, and Fru (Zhang et al., 2018).

### 2.9 Measurement of the production of ethylene and lycopene content

The production of ethylene was measured as previously described (Jia et al., 2017b). Tomato fruits were placed in a 0.86-L sealed container for 1 h (25°C). Subsequently, a syringe was used to collect 1 mL of gas. Agilent 7890A (the gas chromatograph; Santa Clara, CA, USA) was used for ethylene measurement, as previously described (Jia et al., 2017b).

For lycopene content, 0.4 g of fruit tissue was collected and measured as described by Ji et al. (2020).

### 2.10 MY2H

*SlSWEET7a*, *SlSWEET14*, *SlRLK-like*, *SlACS2*, *SlPSY1*, and *SlSAMS4* CDSs with *Hin*dIII sites were constructed into the bait vector (pBT3-STE). *SlRLK-like*, *SlACS2*, *SlPSY1*, and *SlSAMS4* CDSs with the *Kpn*I site were introduced into the prey vector (pPR3-N) using primers in **Supplementary Table S1**. The fused vectors were introduced into the NMY51 yeast strain following Dualsystems Biotech’s instructions. The yeast transformants were then screened using SD/–Leu/–Trp (double dropout agar medium) + X-gal to screen the positive fused yeast clones. X-gal is a specific inhibitor of β-galactosidase, which inhibits the activity of the enzyme, preventing the glycosidase from breaking down β-galactosidase, and when the enzyme is inhibited, X-gal turns into a blue precipitate. SD/–His/–Leu/–Trp/–Ade (quadruple dropout medium) was also used for screening the positive fused yeast clones.

### 2.11 BiFC assay

*SlRLK-like*, *SlSWEET7a*, *SlSWEET14*, *SlACS2*, *SlPSY1*, and *SlSAMS4* CDSs were cloned into the pCAMBIA1300-35S-C-YFPC/YFPN vector using the primers shown in **Supplementary Table S1**. The negative control groups were as follows: cYFP empty vector + nYFP empty vector, SlRLK-like-cYFP + nYFP empty vector, SlRLK-like-nYFP + cYFP empty vector, SlSWEET7a-cYFP + nYFP empty vector, SlSWEET7a-nYFP + cYFP empty vector, SlSWEET14-cYFP + nYFP empty vector, SlSWEET14-nYFP + cYFP empty vector, SlACS2-cYFP + nYFP empty vector, SlACS2-nYFP + cYFP empty vector, SlSAMS4-cYFP + nYFP empty vector, SlSAMS4-nYFP + cYFP empty vector, SlPSY1-cYFP + nYFP empty vector, and SlPSY1-nYFP + cYFP empty vector. The resulting constructs were then co-introduced into *Agrobacterium tumefaciens* strain GV3101 and injected into *N. benthamiana* epidermal cells. Then, fused constructs were co-transformed into GV3101 and co-injected into the epidermal cells of *N. benthamiana* leaves. At 2–3 days after injection, Leica SP8 (confocal laser scanning microscope; Germany) was used to observe the fluorescence signals, with 488-nm excitation wavelength and 500–572-nm emission wavelengths.

### 2.12 Assays of esculin fluorescence in yeast cells

The fluorescent experiments of esculin uptake activity were conducted as reported (Abelenda et al., 2019; Gora et al., 2012). The CDSs of *SlRLK-like*, *SlSWEET7a*, and *SlSWEET14* were introduced into the PDR195 vector and further transformed into Susy7/-ura (a sucrose transport defective yeast strain). The fused strains were cultivated in SD/-ura [with 2% (w/v) Glu] at 30°C for approximately 16 h at 6,000 rpm for 5 min to collect the yeast cells. Then, 1 mL of 250 mM Na_2_HPO_4_ (pH = 3.5) was used to wash them. The positive fused strains were selected via PCR examination. Then, the picked fused strains were cultivated in solid SD/-ura [with 2% (w/v) Glu] at 30°C and 180 rpm for approximately 16 h until OD_600nm_ = 1. Then, the fused strains were incubated in 1 mL of 250 mM Na_2_HPO_4_ (pH = 3.5) with 8 mM esculin for 1 h at 30°C. The cells were washed twice with 1 mL of 250 mM Na_2_HPO_4_ (pH = 3.5). The fluorescence of esculin was measured at 367-nm excitation wavelength and 454-nm emission wavelength.

### 2.13 Statistical analysis

The data in this study were statistically analyzed using the Prism 5 software from GraphPad and expressed as mean ± standard deviation. Either Student’s t-test (*p < 0.05) or a one-way analysis of variance was used for group comparisons. All the experiments performed in this study were repeated at least three times.

## 3 Results

### 3.1 SlSWEET14 interacts with SlRLK-like

**Figure 1:**
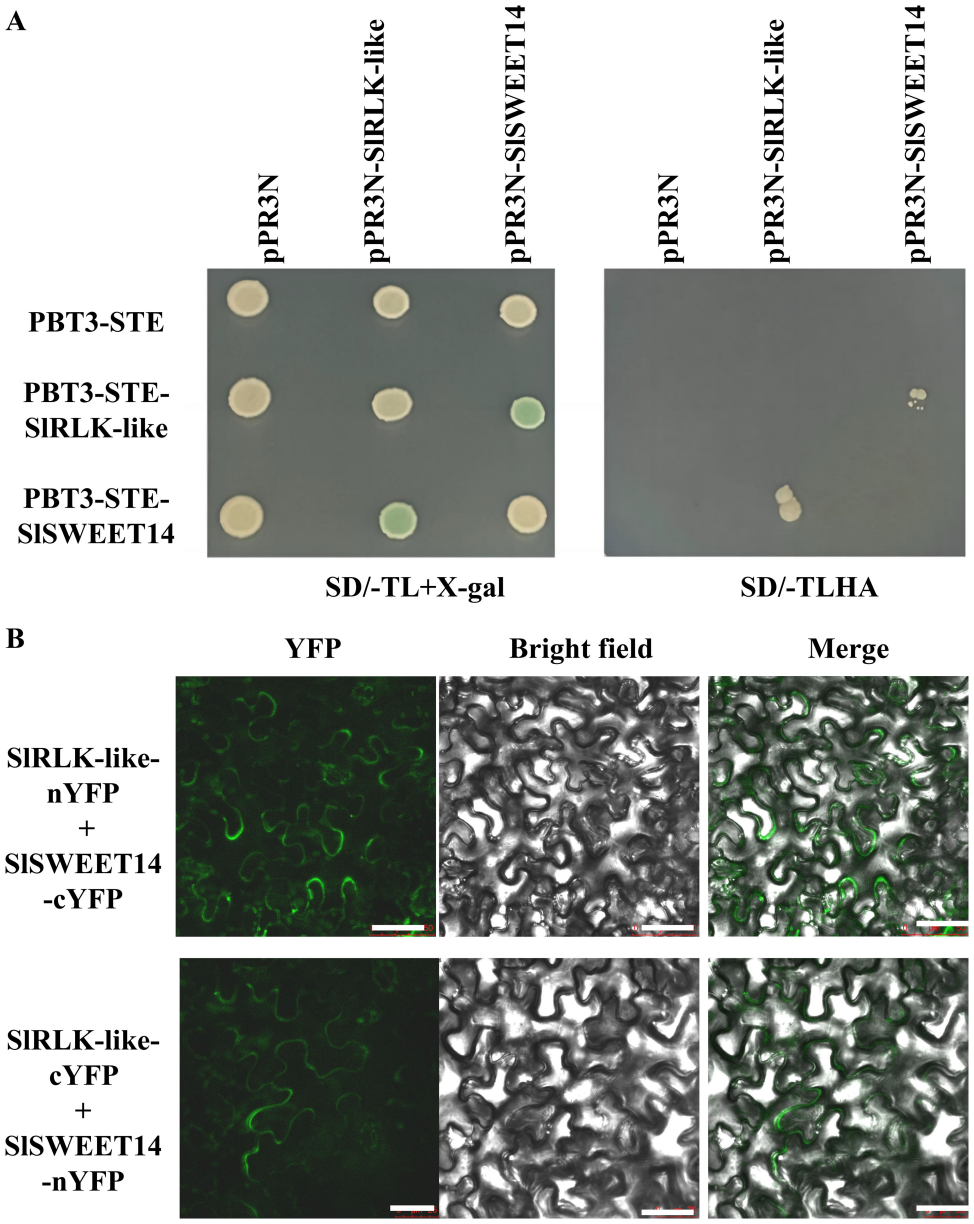
Interaction between SlRLK-like and SlSWEET14. **(A)** MY2H assays of SlRLK-like and SlSWEET14. **(B)** BiFC assays of the interaction between SlRLK-like and SlSWEET14 in *Nicotiana benthamiana* epidermal cells. Scale bars = 25 μm. This experiment was performed at least three times. MY2H, membrane-based yeast two-hybrid; BiFC, bimolecular fluorescence complementation.

SlRLK-like was identified using the membrane-based yeast two-hybrid methodology, with tomato fruit cDNA libraries and SlSWEET14 serving as the prey and the bait, respectively (Zhang et al., 2024). Among several potential proteins, SlRLK-like (Solyc01g094920) was selected, as it had the highest hit number (**Table 1**). Membrane-based yeast two-hybrid (MY2H), together with the interactions between SlRLK-like and SlSWEET14, was verified by bimolecular fluorescence complementation (BiFC) assays and MY2H (**Figures 1A,B**; **Supplementary Figure S1**). SlSWEET14 interacted with SlRLK-like and was colocalized on the plasma membrane. SlRLK-like is a plasma membrane-located protein and belongs to the M/MLD-RLK family (Sussholz et al., 2020), and SlSWEET14 was also reported as a plasma membrane-located protein (Zhang et al., 2021b). Phylogenetic tree analysis with *Arabidopsis* confirmed that SlRLK-like also belongs to the THE subfamily (**Supplementary Figure S2**). The interactions between SlRLK-like and SlSWEET14 suggest that SlRLK-like is likely involved in sugar accumulation and tomato fruit ripening. Therefore, we paid more attention to characterizing the function of SlRLK-like in fruits.

**Table 1:** 

Accession number	Annotation	Number of hits
Solyc01g094920	probable receptor-like protein kinase At5g24010	7
Solyc06g072970	zinc finger with UFM1-specific peptidase domain protein isoform X2	4
Solyc07g063100	proton pump-interactor 1	4
Solyc07g047970	SEC12-like protein 2	2
Solyc01g100690	uncharacterized protein LOC101252823	2
Solyc10g054560	V-type proton ATPase 16 kDa proteolipid subunit	2
Solyc04g009550	protein TONNEAU 1a	2
Solyc03g097580	bidirectional sugar transporter N3	1
Solyc07g062700	sodium/calcium exchanger NCL	1
Solyc08g077290	transcription factor MAMYB	1

### 3.2 *SlRLK-like* is highly expressed in tomato fruit

**Figure 2:**
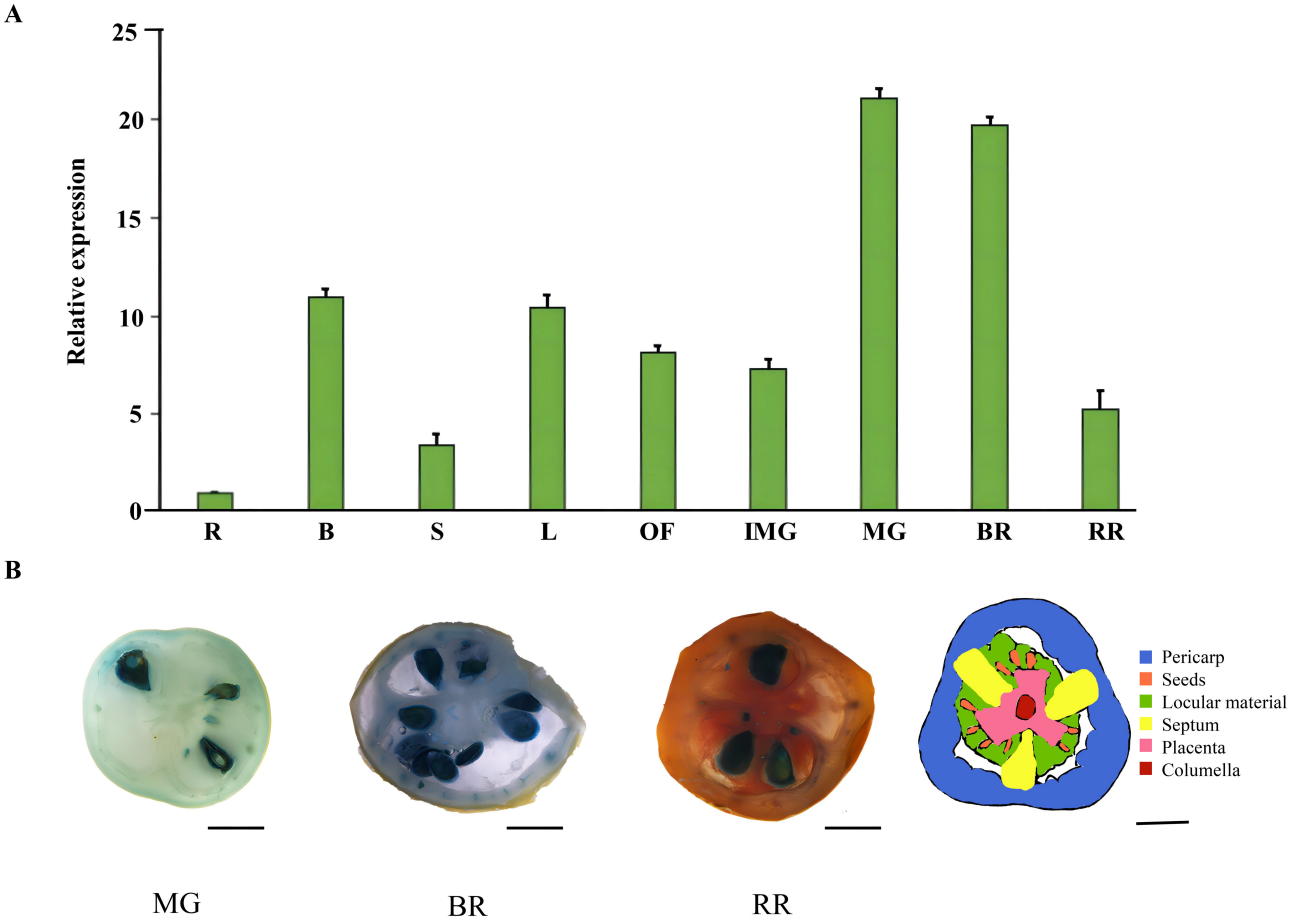
Expression pattern of *SlSlRLK-like* in tomatoes. **(A)** Relative expression level of *SlRLK-like* in different tomato fruit tissues and ripening stages. R, root; B, branch; S, stem; L, source leaves; OF, opened flower. **(B)** GUS stain of SlSlRLK-like. MG (mature green stage), BR (breaker stage), and RR (red ripening stage) are shown from left to right. Scale bars = 1 cm.

The expression patterns of the *SlRLK-like* genes in various tissues were tested via RT-qPCR. The results showed that *SlRLK-like* was highly expressed in most tomato tissues. Notably, during fruit development, *SlRLK-like* was upregulated from IMG to MG, while exhibiting slightly lower expression in fruit during the BR period. During the RR stage, relative expression levels were markedly decreased (**Figure 2A**). Similarly, *SlSWEET14* from clave III was highly expressed during the MG stage (Zhang et al., 2021b). Another SWEET clade II family member, namely, *SlSWEET7a* (Zhang et al., 2021b), exhibits similarly high expression levels as during the MG stage (**Supplementary Figure S3**).

Furthermore, SlRLK-like-GUS fusion proteins expressed in transgenic plants were used to observe the expression levels of SlRLK-like during the development stages of tomato fruits (**Figure 2B**). In MG fruits, SlRLK-like-GUS protein mostly accumulated in seed coats. For vascular tissues of the pericarp and placenta, there was moderate accumulation. GUS activity was found in all pericarp cells in BR fruits, in addition to the seed coat and vascular tissues. In RR fruits, the vascular tissues of the placenta or pericarp had moderate levels of GUS activity, while the seed coats still had high levels. The SlRLK-like was closely linked to sugar unloading in fruits and throughout seed development by the significant accumulation of the GUS fusion protein in vascular tissues and seed coats.

### 3.3 SlRLK-like interacts with SlSWEET7a

**Figure 3:**
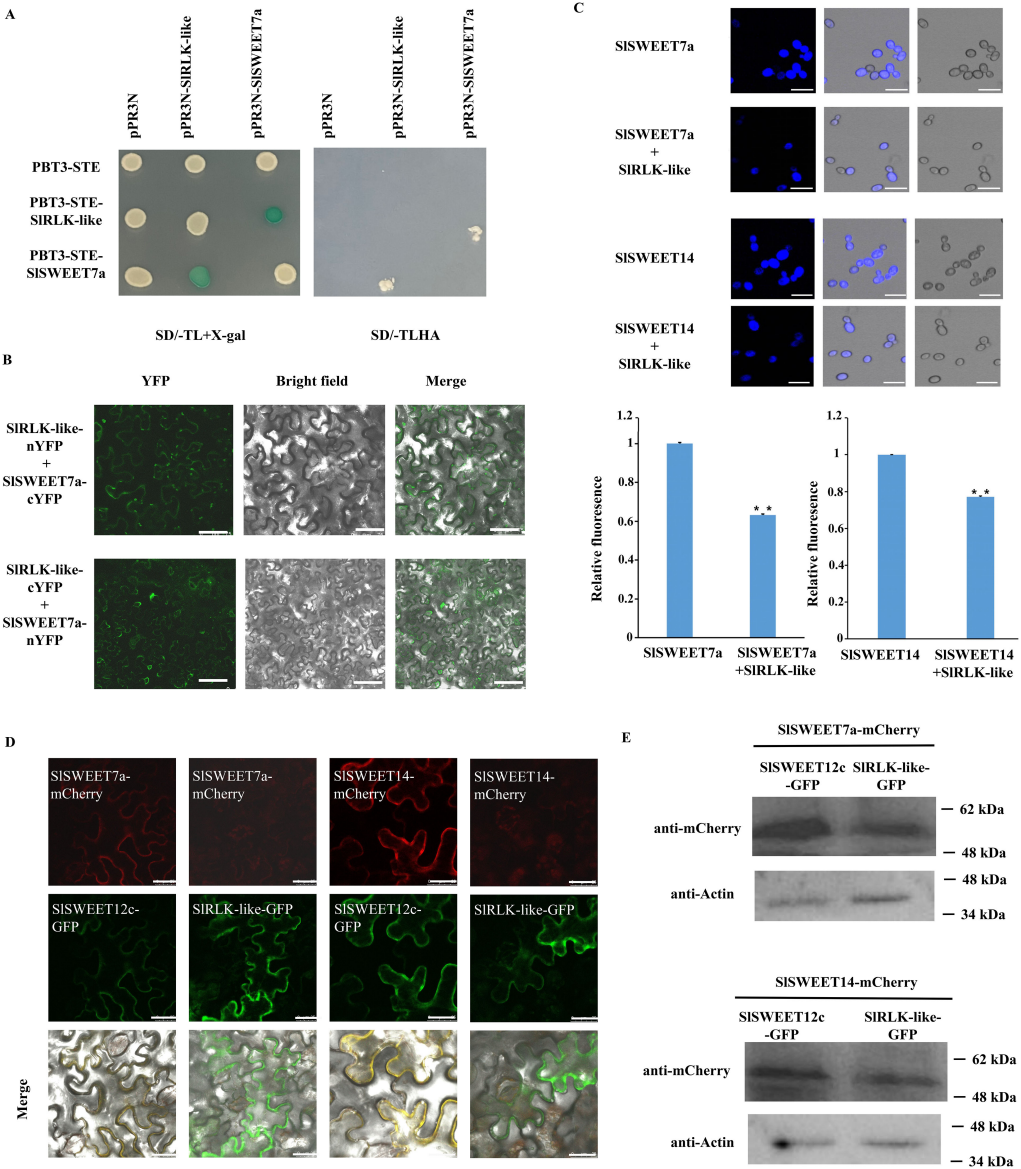
Interaction analysis of SlRLK-like with SlSWEETs. **(A)** MY2H assays of SlRLK-like with SlSWEET7a. **(B)** BiFC assays for the interaction between SlRLK-like and SlSWEET7a in *Nicotiana benthamiana* epidermal cells. Scale bars = 25 μm. **(C)** Fluorescence microscopy assays of susy7/-ura yeast esculin uptake in SlRLK-like, SlSWEET7a, and SlSWEET14. Scale bars = 25 μm. **(D)** Co-expression assays of SlSWEET12c with SlRLK-like, SWEET7a, or SWEET14. Scale bars = 25 μm. Co-expression assays of SlRLK-like with SlSWEET12c, SWEET7a, or SWEET14. Scale bar = 25 µm. **(E)** Western blotting. These experiments were performed at least three times. MY2H, membrane-based yeast two-hybrid; BiFC, bimolecular fluorescence complementation.

Considering the similar expression patterns of *SlRLK-like* and *SlSWEET7a*, their interactions were further verified. The MY2H and BiFC assays revealed that SlRLK-like interacted with SlSWEET7a (**Figures 3A, B**; **Supplementary Figure S1**). Collectively, SlRLK-like interacted with SlSWEET7a and SlSWEET14, suggesting that SlRLK-like participates in sugar accumulation induced by SWEETs in tomato fruit development. To figure out whether the interaction between SlRLK-like and SlSWEETs has a direct effect on sucrose transport, esculin (a fluorescent sucrose analog) and Susy7/-ura (a yeast mutant strain) were used to detect SlRLK-like-mediated sucrose transport activity (**Figure 3C**). When SlSWEET14 and SlSWEET7a were co-expressed with SlRLK-like, the fluorescence was significantly decreased. The above results suggested that SlRLK-like plays a critical part in the transport activity of SlSWEET14 and SlSWEET7a.

To determine whether the abundance and localization of SWEET7a and SWEET14 will change if co-expressed with SlRLK-like, SlRLK-like-GFP with SWEET7a-mCherry and SWEET14-mCherry were co-expressed in *N. benthamiana* epidermal cells, respectively. SWEET12c-mCherry/-GFP, which was proven to be located on the plasma membrane (Sun et al., 2022), was used as the control. Co-expression results showed that when SlSWEET12c-GFP was co-expressed with SWEET7a-mCherry or SWEET14-mCherry, they co-localized on the plasma membrane (**Figure 3D**). However, the co-expression between SlRLK-like-GFP and SWEET7a-mCherry or SlRLK-like-GFP and SWEET14-mCherry caused the dispersion in red signal (**Figure 3D**). Western blotting was used to measure the expression levels of SWEET7a and SWEET14 in co-expressing *N. benthamiana* epidermal cells. The results showed that when co-expressed with SlRLK-like, the expression levels of SWEET7a and SWEET14 were decreased (**Figure 3E**). Taken together, these results indicate that SlRLK-like regulated the transport activity of SWEETs by modulating their abundance and localization.

### 3.4 SlRLK-like regulates tomato fruit sugar storage

**Figure 4:**
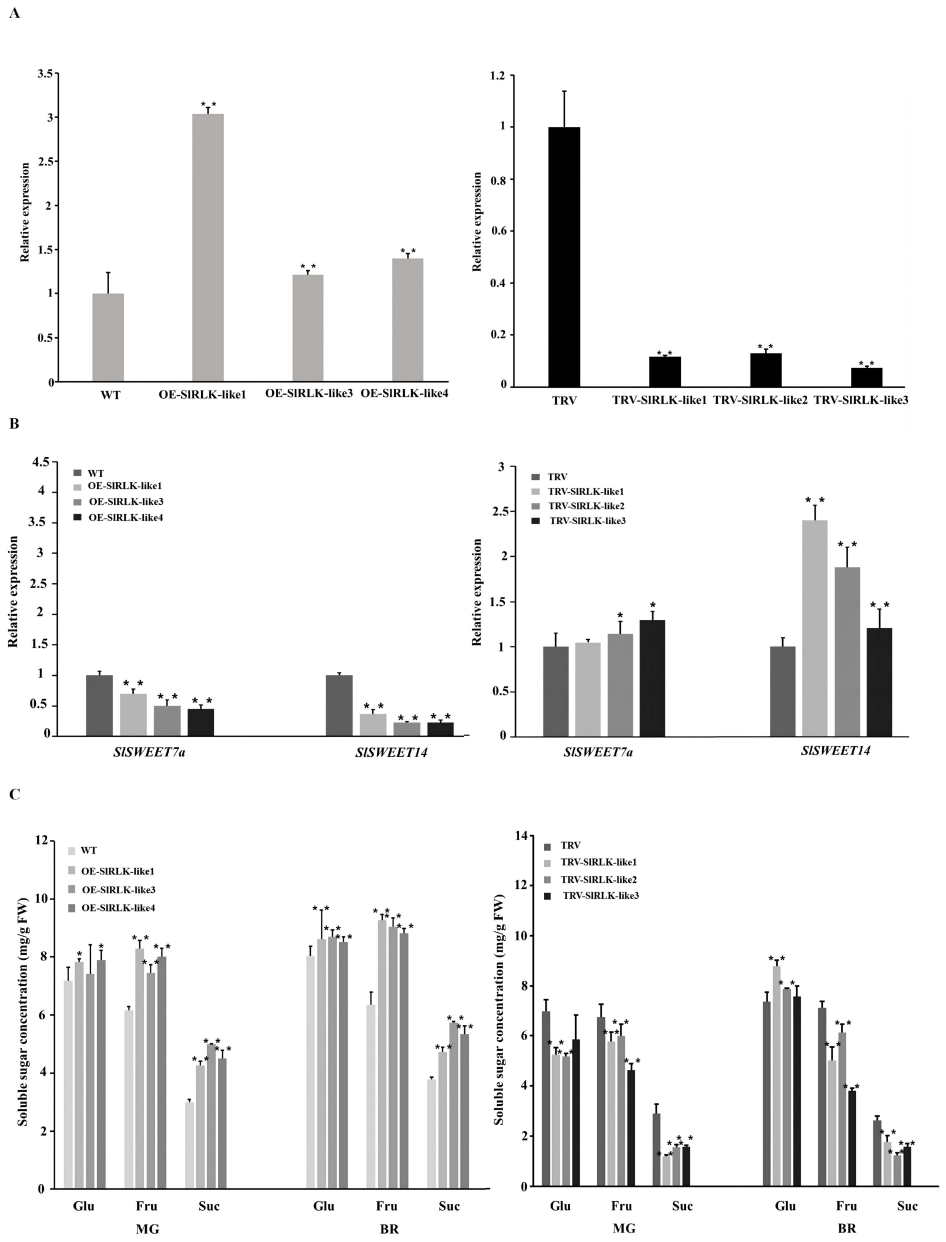
Overexpression (OE) or silencing of *SlRLK-like* alters sugar accumulation in tomato fruits. **(A)** Expression level of *SlRLK-like* in WT, OE-*SlRLK-like* T_3_ fruits, and VIGS fruits. **(B)** Expression level of *SlSWEETs* in OE-*SlRLK-like* and TRV-*SlRLK-like* lines. **(C)** Sugar concentration in WT and transgenic plants. This experiment was performed at least three times; *p < 0.05 and **p < 0.01. VIGS, virus-induced gene silencing; WT, wild type.

To determine the physiological functions of SlRLK-like proteins, OE transgenic lines were constructed as the T_3_ generation; VIGS lines were also obtained. According to the RT-qPCR results, three OE-*SlRLK-like* lines and three TRV-*SlRLK-like* plants were selected for further analysis (**Figure 4A**). *SlSWEET7a* and *SlSWEET14* relative expression levels in the OE-*SlRLK-like* and TRV-*SlRLK-like* lines differed significantly (**Figure 4B**). During the MG stage, *SlWEET7a* and *SlSWEET14* were observably down- and upregulated in OE-*SlRLK-like* and SlRLK-silenced fruits, respectively.

The role that SlRLK-like plays in tomato fruit sugar storage was also further investigated, and the concentrations of soluble sugars were assayed (**Figure 4C**). Fru concentration in OE-*SlRLK-like* MG fruits was 20%–30% higher than that in WT fruits. Furthermore, the Suc content increased by 42%–66% compared to that in WT fruits. Meanwhile, the Glu concentration showed no significant changes. Regarding the TRV-*SlRLK-like* MG fruits, the concentrations of Glu, Fru, and Suc decreased by 16%–25%, 11%–31%, and 44%–58%, respectively, compared to those in the control lines. During the BR stage, OE-*SlRLK-like* fruits contained ~38%–45% more Fru than the WT. The Suc concentration also increased by an estimated 25%–52%, compared to that in WT. At this stage, the Glu content in the OE-*SlRLK-like* fruits also increased. In contrast, the Fru and Suc contents decreased significantly in the *SIRLK-like*-silenced lines. However, the content of Glu was increased by 2%–19% compared to that in the control. Considering the impact of fruit weight on fruit sugar content, the fruit weights of OE-*SlRLK-like*, TRV-*SlRLK-like*, and control lines during the MG stage were measured (**Supplementary Figure S4**). The results showed that overexpressing *SlRLK-like* caused a significant increase in fruit weight. There was no significant difference in the fruit weight between TRV and TRV-*SlRLK-like* lines. These results suggest that SlRLK-like promotes Fru and Suc accumulation during fruit ripening.

### 3.5 SlRLK-like modulates the fruit ripening process in tomatoes

**Figure 5:**
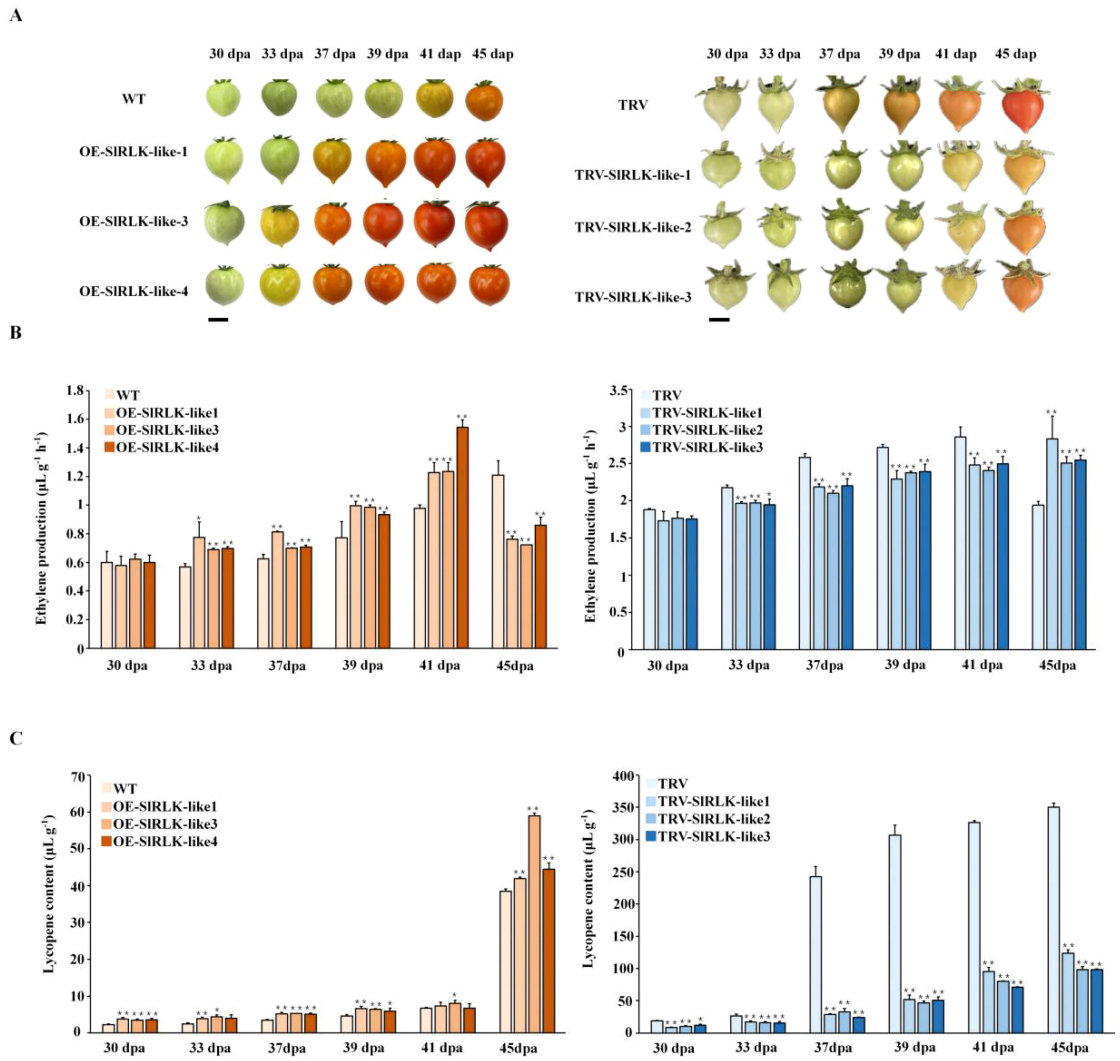
Overexpression (OE) or silencing of *SlRLK-like* alters fruit ripening progress in tomatoes. **(A)** Ripening phenotype of WT and transgenic fruits at 30, 33, 37, 39, 41, and 45 dpa. Scale bars = 1 cm. **(B)** The ethylene production of WT and transgenic lines at 30, 33, 37, 39, 41, and 45 dpa. **(C)** Lycopene content of WT and transgenic lines at 30, 33, 37, 39, 41, and 45 dpa. This experiment was performed at least three times; *p < 0.05 and **p < 0.01. WT, wild type; dpa, days post-anthesis.

During tomato fruit ripening, OE-*SlRLK-like* lines ripened much earlier than the WT (**Figure 5A**). Thirty days post-anthesis, both WT and OE-*SlRLK-like* fruits were in the mature green stage. However, at 33 dpa, the OE-*SlRLK-like* fruits began to turn orange, while WT fruits remained green. In OE-*SlRLK-like* lines, the period from the MG stage to the BR stage was accelerated by ~5–8 days compared to that in WT lines. In TRV-*SlRLK-like* fruits, the ripening process was reduced (**Figure 5A**). TRV-*SlRLK-like* fruits reached the BR stage at 41 dpa, whereas control TRV fruits turned orange at 37 dpa; the period of this was delayed by 4–5 days. To explore these changes, ethylene production and lycopene content were investigated (**Figures 5B, C**). Ethylene production and lycopene content in OE-*SlRLK-like* fruits were markedly higher than those in the WT fruits. Furthermore, compared to those in the TRV control fruits, ethylene production and lycopene content in TRV-*SlRLK-like* fruits were significantly decreased. Ethylene production in OE-*SlRLK-like* fruits was increased by 20%–60% from 30 to 41 dpa compared with that in WT fruits. At 41 dpa, ethylene production in OE-*SlRLK-like* fruits peaked much earlier than in WT fruits. The peak ethylene production in WT fruits was delayed by 4 days compared to that in OE-*SlRLK-like* fruits. Meanwhile, in TRV-*SlRLK-like* fruits, ethylene production was decreased by 10%–20% compared to that in the TRV control fruit. Furthermore, the ethylene peak in TRV-*SlRLK-like* lines was delayed by at least 4 days compared to that in the TRV control lines. These changes suggest that SlRLK-like acts as a novel regulator in the tomato fruit ripening process.

### 3.6 SlRLK-like influences ethylene production and lycopene accumulation

**Figure 6:**
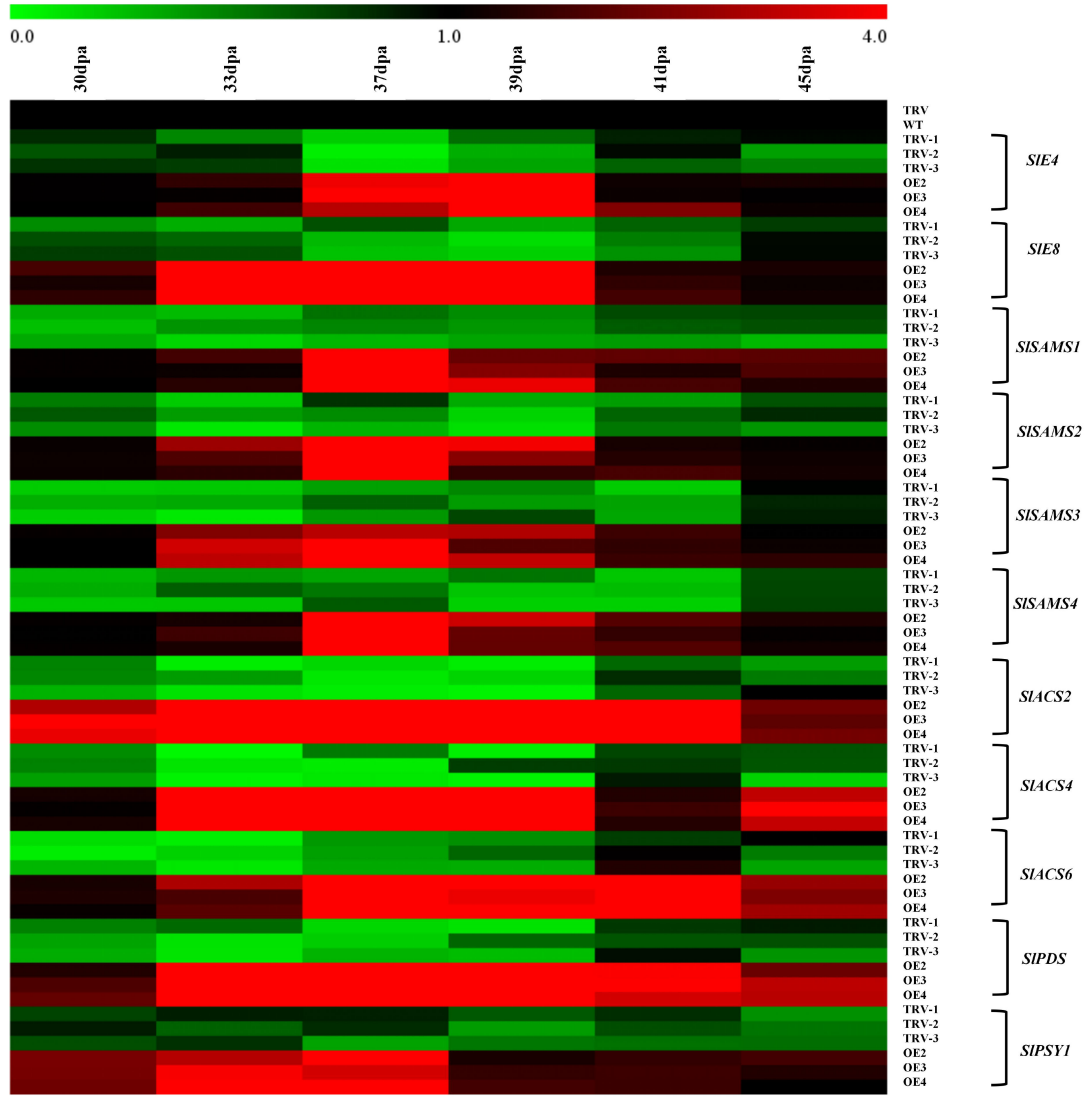
RT-qPCR assays of ethylene- and lycopene biosynthesis-related genes. Internal control: *SlACTIN*. Primers are shown in **Supplementary Table S1**. Fruits of WT and transgenic were harvested at 30, 33, 37, 39, 41, and 45 dpa. Similar experiments were repeated at least three times. WT, wild type; dpa, days post-anthesis.

Considering the role of *SlRLK-like* in modulating the process of tomato fruit ripening, the transcription levels of ripening-associated, ethylene biosynthesis, and lycopene accumulation genes were analyzed (**Figure 6**). It was found that there were significant up- and downregulation in *E4* and *E8* (ripening-associated genes) expression in OE-*SlRLK-like* and TRV-*SlRLK-like* lines, respectively, compared to the control lines. Ethylene biosynthesis-related genes, including *SlSAMS1*–*4*, *SlACS2*, *SlACS4*, and *SlACS6*, exhibited the same expression patterns as *E4* and *E8*, all of which increased or decreased in OE-*SlRLK-like* and TRV-*SlRLK-like* fruits, respectively, compared to control fruits during fruit ripening. *PSY1* and *PDS* (lycopene biosynthesis-associated genes) were both up- and downregulated in OE-*SlRLK-like* and TRV-*SlRLK-like* lines, respectively. Collectively, these results show that SlRLK-like regulates tomato fruit ripening by modulating the expression of genes, which were involved in ethylene production and lycopene accumulation.

### 3.7 SlRLK-like interacts with SlSAMS4, SlACS2, and SlPSY1

**Figure 7:**
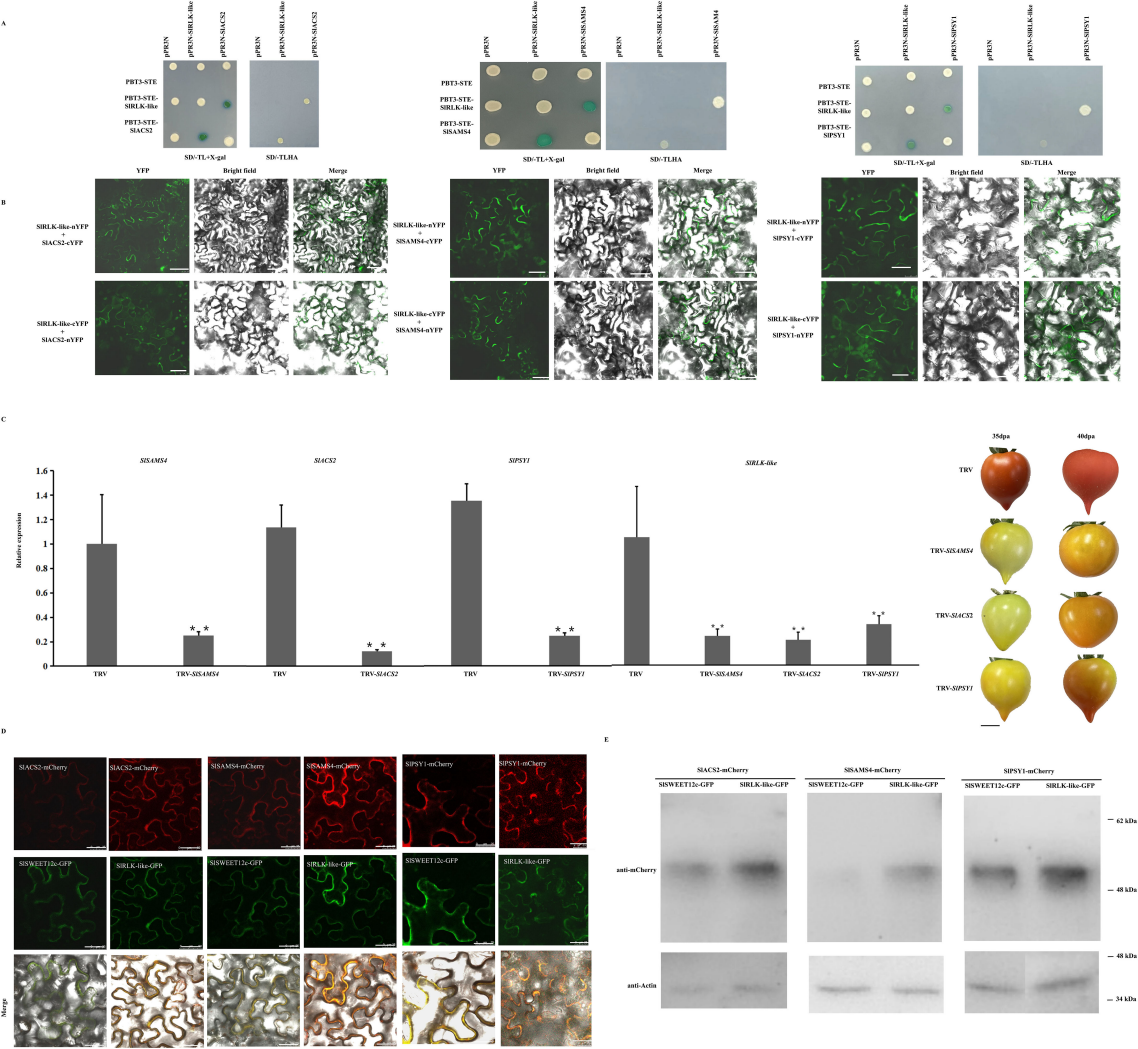
Interaction analysis of SlRLK-like with SlACS2, SlSAMS4, and SlPSY1. **(A)** MY2H assays of interaction between SlRLK-like with SlACS2, SlSAMS4, and SlPAY1. **(B)** BiFC assays for the interaction between SlRLK-like with SlACS2, SlSAMS4, and SlPSY1 in *Nicotiana benthamiana* epidermal cells. Scale bars = 25 μm. **(C)** The *SlSAMS4*, *SlACS2*, *SlPSY1*, and *SlRLK-like* expression in TRV transgenic fruits. Tomato fruit ripening phenotype in TRV-*SlSAMS4*, TRV-*SlACS2*, and TRV-*SlPSY1* fruits at 35 and 40 dpa. Scale bar = 1 cm. **(D)** Co-expression assays of SlSWEET12c with SlRLK-like, SAMS4, ACS2, or PSY1. Co-expression assays of SlRLK-like with SlSWEET12c, SAMS4, ACS2, or PSY1. Scale bar = 25 µm. **(E)** Western blotting. This experiment was performed at least three times; *p < 0.05 and **p < 0.01. MY2H, membrane-based yeast two-hybrid; BiFC, bimolecular fluorescence complementation; dpa, days post-anthesis.

Considering the differences in ripening-related gene expression between OE-*SlRLK-like* and TRV-*SlRLK-like* fruits, the physical interactions between these enzymes were further examined during ethylene and lycopene biosynthesis. As SlRLK-like is a plasma membrane-located protein, the split ubiquitin MY2H was used for analysis (**Figure 7A**). MY2H results showed that SlRLK-like proteins interacted with SlSAMS4, SlACS2, and SlPSY1; this interaction was confirmed in plants using BiFC (**Figure 7B**; **Supplementary Figure S1**). When SlRLK-like-nYFP and SlACS2-cYFP, SlRLK-like-nYFP and SlSAMS4-cYFP, and SlRLK-like-nYFP and SlPSY1-cYFP were used, green fluorescence was observed in *N. benthamiana* leaves. Hence, SlRLK-like interacted with SlSAMS4, SlASC2, and SlPSY1 in the plasma membrane.

We also identified the roles that SlACS2, SlSAMS4, and SlPSY1 play in the ripening stage of tomato fruits. That is, VIGS lines for *SlACS2*, *SlSAMS4*, and *SlPSY1* with markedly downregulated expression of their mRNA were created (**Figure 7C**). Similarly, *SlRLK-like* transcript levels were downregulated (**Figure 7C**). TRV-*SlACS2*, TRV-*SlSAMS4*, and TRV-*SlPSY1* fruits all showed a much slower ripening process than control fruits (**Figure 7C**). These results suggest that SlACS2, SlSAMS4, and SlPSY1 positively regulate tomato fruit ripening.

Moreover, the co-expression with SlRLK-like-GFP did not change the localization of SAMS4, ACS2, and PSY1; also, the red signals were enhanced (**Figure 7D**). Using Western blotting, we found that the expression levels of SAMS4, ACS2, and PSY1 were increased when co-expressed with SlRLK-like in *N. benthamiana* epidermal cells (**Figure 7E**). Altogether, SlRLK-like modulated tomato fruit ripening by regulating SAMS4, ACS2, and PSY1 abundance and the signal strength of localization.

## 4 Discussion

### 4.1 SlRLK-like impacts sugar accumulation by interacting with SlSWEETs during tomato fruit development

One important criterion for tomato fruit quality is its sugar concentration, with sugar accumulation being a highly coordinated process requiring various metabolic enzymes and sugar transporters. During this process, monosaccharides and disaccharides are passively transported across membranes via concentration gradients by SWEETs (Chen et al., 2010). However, considering their seven transmembrane domains, oligomerization is vital for SWEET transport (Xuan et al., 2013; Xu and Liesche, 2021). CsSWEET1a interacts with CsSWEET17 to form homo-/heterodimers in the plasma membrane to regulate sugar allocation between the cytoplasm and apoplast (Yao et al., 2020). Meanwhile, SlSWEET14 regulates the transport and accumulation of sugar in tomato fruits by interacting with SlSWEET7a (Zhang et al., 2021b). Apart from oligomerization, another way that post-translational SWEET regulation occurs is through interactions with other proteins. StSP6A, a transcription factor homologue of the flowering locus, interacts with StSWEET11 in potatoes to prevent Suc export to the apoplast, hence encouraging symplastic Suc unloading and starting the creation of potato tubers (Abelenda et al., 2019). In addition, a previous study showed that SlRLK-like does not have a kinase domain. It functions by interacting with downstream proteins and regulating their protein abundance (Sussholz et al., 2020). In our study, SlRLK-like was chosen because it is the most potent protein in the MY2H system. Based on the expression pattern and interaction assays, we found that SlRLK-like interacted with SlSWEET7a and SlSWEET14 (**Figures 1**, **3**; **Supplementary Figure 1**). The sucrose transport activity of SlSWEETs was also changed when they interacted with SlRLK-like (**Figure 3C**). Co-expression with SlRLK-like almost made SlSWEETs undetectable in yeast cells (**Figure 3C**). Meanwhile, the overexpression or silencing of *SlRLK-like* altered sugar accumulation during tomato fruit development (**Figure 4C**). A previous study also showed that *SlSWEET14* and *SlSWEET7a* RNAi fruit accumulated more Fru and Glu than WT fruits during the MG stages, which was similar to our result (Zhang et al., 2021b). Meanwhile, SlRLK-like also seemed to regulate the tomato fruit weight (**Supplementary Figure S4**). However, the change in fruit sugar content between OE-*SlRLK-like* and TRV-*SlRLK-like* was not affected by fruit weight, which suggested that SlRLK-like regulated fruit sugar accumulation in an individual pathway.

In strawberries, the overexpression or RNAi silencing of *FaMRLK47* promotes and inhibits the expression of *FaSS* and *FaSPS1* (genes involved in sucrose biosynthesis), respectively. The sugar (Fru, Glu, and Suc) content was also significantly increased in RNAi-*FaMRLK47*, while the Fru and Suc contents were reduced in RNAi-*FaMRLK47* fruits. These results suggest that FaMRLK47 promotes the hydrolysis of Suc (Jia et al., 2017a). In our study, SlRLK-like interacted with SlSWEET14 and SlSWEET7a, while both *SlSWEET7a* and *SlSWEET14* expression levels in OE-*SlRLK-like* fruits were significantly downregulated compared with those in WT (**Figure 4C**). The change in sugar accumulation in OE-*SlRLK-like* fruit was similar to that observed in *SlSWEET7a* and *SlSWEET14* RNAi fruits. Overall, SlRLK-like was found to modulate sugar accumulation by interacting with SlSWEETs.

### 4.2 SlRLK-like functions as a positive tomato fruit ripening regulator

The ripening physiological process of fleshy fruits is complex and associated with alterations in aroma, color, flavor, and hardness. Tomatoes are climacteric fruits and an excellent fleshy fruit model. Accordingly, in order to improve the quality of tomato fruits, it is important to figure out the regulation mechanism in tomato ripening (Pesaresi et al., 2014). Ethylene is a key plant hormone controlling the ripening of climacteric fruit. Ethylene synthesis, perception, and responses in plant ethylene signal transduction influence climacteric fruit ripening (Liu et al., 2015). FER serves as the first-function M/MLD-RLK member, while SlFERL—an AtFER homologue—interacts with SlSAMS1 to positively regulate tomato fruit ripening (Ji et al., 2020). SlRLK-like can reportedly interact with LeEIX2 (a fungal elicitor) and negatively regulate defense reactions by binding to xylanase, which was induced by ethylene. With EIX induced, ethylene production was decreased and increased in OE-*SlRLK-like* and function-loss *SlRLK-like* leaves, respectively. Furthermore, *SlACS2* was upregulated and expressed in function-loss *SlRLK-like* leaves when induced by EIX (Sussholz et al., 2020). Our study indicated that ethylene production and lycopene content also increased and decreased in OE-*SlRLK-like* and TRV-*SlRLK-like* fruits, respectively (**Figure 5B**).

As a precursor to molecular changes that affect the fruit’s color, flavor, texture, aroma, and nutritional qualities, the respiratory peak in tomato fruits and ethylene bursts take place at the start of ripening (Lü et al., 2018; Huang et al., 2023). In the present study, we found that *SlRLK-like* was abundantly expressed during tomato fruit ripening (**Figure 2**). In addition, the peak of ethylene production occurred in OE-*SlRLK-like* and TRV-*SlRLK-like* fruits much earlier and later, respectively, than in the control fruits (**Figures 5A, B**).

The ethylene biosynthesis pathway begins with SAMS, which converts methionine to SAM, followed by ACS converting SAM to ACC. ACO then oxidizes ACC into ethylene (Liu and Zhang, 2004). SAM, as an intermediate metabolite between ACC and Met, has the ability to control the production of ethylene. Additionally, SAMS plays an important role at this stage. In rice, OsSAMS1 interacts with Pns11—a protein-encoding the rice dwarf virus—thereby improving the susceptibility between rice seedlings and the rice dwarf virus, enhancing the enzymatic activity of OsSAMS1 and improving SAM, ACC, and ethylene production (Zhao et al., 2017). Furthermore, in *Arabidopsis*, AtFER negatively modulates SAM level and ethylene biosynthesis by interacting with SAM synthetase (Mao et al., 2015). Oppositely, SlFERL positively regulates tomato fruit ripening and ethylene biosynthesis by interacting with SlSAMS1 in tomatoes (Ji et al., 2020). In tomatoes, *SlSAMS1*, *SlSAMS2*, *SlSAMS3*, and *SlSAMS4* contribute to fruit development (Ji et al., 2020; Van et al., 2013). Within the current study, they were all significantly upregulated in OE-*SlRLK-like* fruits compared to the WT lines. However, in the TRV-*SlRLK-like* lines, *SlSAMSs* was downregulated, compared to the control fruits (**Figure 6**). ACS2 and ACS4 are the other vital enzymes that are involved in both fruit ripening and the ethylene biosynthesis process (Kamiyoshihara et al., 2010). SlMPK3 phosphorylates SlACS2 to modulate ethylene production (Zhou et al., 2023). *SlACS2*, *SlACS4*, and *Sl*ACS6 were all upregulated and downregulated in OE-*SlRLK-like* and TRV-*SlRLK-like* fruits, respectively, compared to the control lines (**Figure 6**). In this study, we confirmed that SlRLK-like did not interact with SlACS4 or SlACS6. Hence, the upregulated expression of *SlACS4* and *SlACS6* may have been caused by the feedback regulation of the ethylene biosynthesis pathway. These results suggest that SlRLK-like takes part in the ripening process of tomato fruit by regulating the production of ethylene.

Color change is another important indicator of fruit ripening. In tomatoes, the lycopene produced by carotenoid synthesis is a key factor that determines ripeness (Shinozaki et al., 2018). Meanwhile, PSY and PSD have important roles in lycopene composition (Lu and Zhu, 2022). *SlPSY1*—a PSY-encoding gene—is expressed in tomatoes. Furthermore, *SlPSY1* is directly related to lycopene accumulation and regulates tomato fruit color (Osorio, 2019). Our study showed that the speed of color change was markedly higher in OE-*SlRLK-like* fruits than in WT fruits (**Figure 5A**). In addition, OE-*SlRLK-like* fruits accumulated more lycopene than the WT fruits from 30 to 45 dpa (**Figure 5C**). Moreover, *PSD* and *PSY*1 were significantly upregulated in the OE-*SlRLK-like* fruits. However, the TRV-*SlRLK-like* lines exhibited opposite results (**Figure 6**). Overall, SlRLK-like was found to be associated with lycopene accumulation, functioning as a positive regulator to improve lycopene content in tomato fruits. Furthermore, SlRLK-like interacted with SlSAMS1, SlAC2, and SlPSY1 (**Figures 7A, B**; **Supplementary Figure S1**), whereas silencing *SlSAMS1*, *SlAC2*, or *SlPSY1* downregulated the expression of SlRLK-like and delayed ripening (**Figure 7C**). Co-expressing SlRLK-like with SlSAMS4, SlACS2, or SlPSY1 did not change the localization of SlSAMS4, SlACS2, or SlPSY1. Moreover, the red signal of SlSAMS4, SlACS2, or SlPSY1 was enhanced (**Figure 7D**). The co-expression with SlRLK-like also made the protein expression levels of SlSAMS4, SlACS2, or SlPSY1 enriched (**Figure 7E**).

Tomato fruit ripening is a complex biological process involving texture softening, color transformation, and the synthesis of flavor substances, which directly affects nutritional quality and commercial value (Mubarok et al., 2023). This process is regulated in coordination by ethylene signaling and multi-level transcriptional networks (such as core transcription factors like HY5, RIN, and FUL1). Recent studies have revealed that these factors shape fruit quality by regulating the metabolic networks of flavonoids, solanine, and glycolic acid (Niu et al., 2025). The regulation of fruit ripening mechanisms focuses not only on hormones (mainly ethylene) but also on the demethylation of key genes, which alters their transcriptional levels, thereby initiating and propagating a cascade of physiological events (Zhou et al., 2024). Additionally, phosphorylation or dephosphorylation also modulates the ripening process of tomato fruits (Li et al, 2024). Here, we found that SlRLK-like, a kinase domain lacking RLK, can also be involved in regulating the tomato fruit ripening process. Unlike other TFs and proteins, SlRLK-like interacted with lycopene- and ethylene-related proteins (SlSAMS1, SlAC2, and SlPSY1) by altering their gene transcription and protein abundance to regulate the biosynthesis of both lycopene and ethylene, which further regulated the ripening process of tomato fruits. Our work provides a new target for molecular mechanisms in regulating tomato fruit quality and ripening via the control of gene and protein interactions.

### 4.3 SlRLK-like increases Suc content in tomato fruit and improves ethylene production

Sugars, especially Suc, promote fruit ripening by improving ethylene biosynthesis. *ACS2*, *ACS4*, and *ACO1* expression levels are regulated via Suc (Hong et al., 2004; Li et al., 2016). In our study, the overexpression of *SlRLK-like* resulted in significantly higher levels of Suc and Fru accumulation in MG and BR fruits (**Figure 4C**). However, the contents of Suc and Fru were significantly decreased in TRV-*SlRLK-like* MG and BR fruits (**Figure 4C**). Considering that Suc enhances ethylene biosynthesis and signal transduction (Li et al., 2016), the differences in ripening traits between OE-*SlRLK-like* and TRV-*SlRLK-like* fruits may have been caused by Suc accumulation. In contrast, Glu negatively regulates ethylene production (Hong et al., 2004). Meanwhile, ethylene production in OE-*SlRLK-like* fruits peaked at 41 dpa (**Figure 5B**). Overall, these results indicate that SlRLK-like increases sugar accumulation, especially Suc, which may improve ethylene production.

## 5 Conclusions

**Figure 8:**
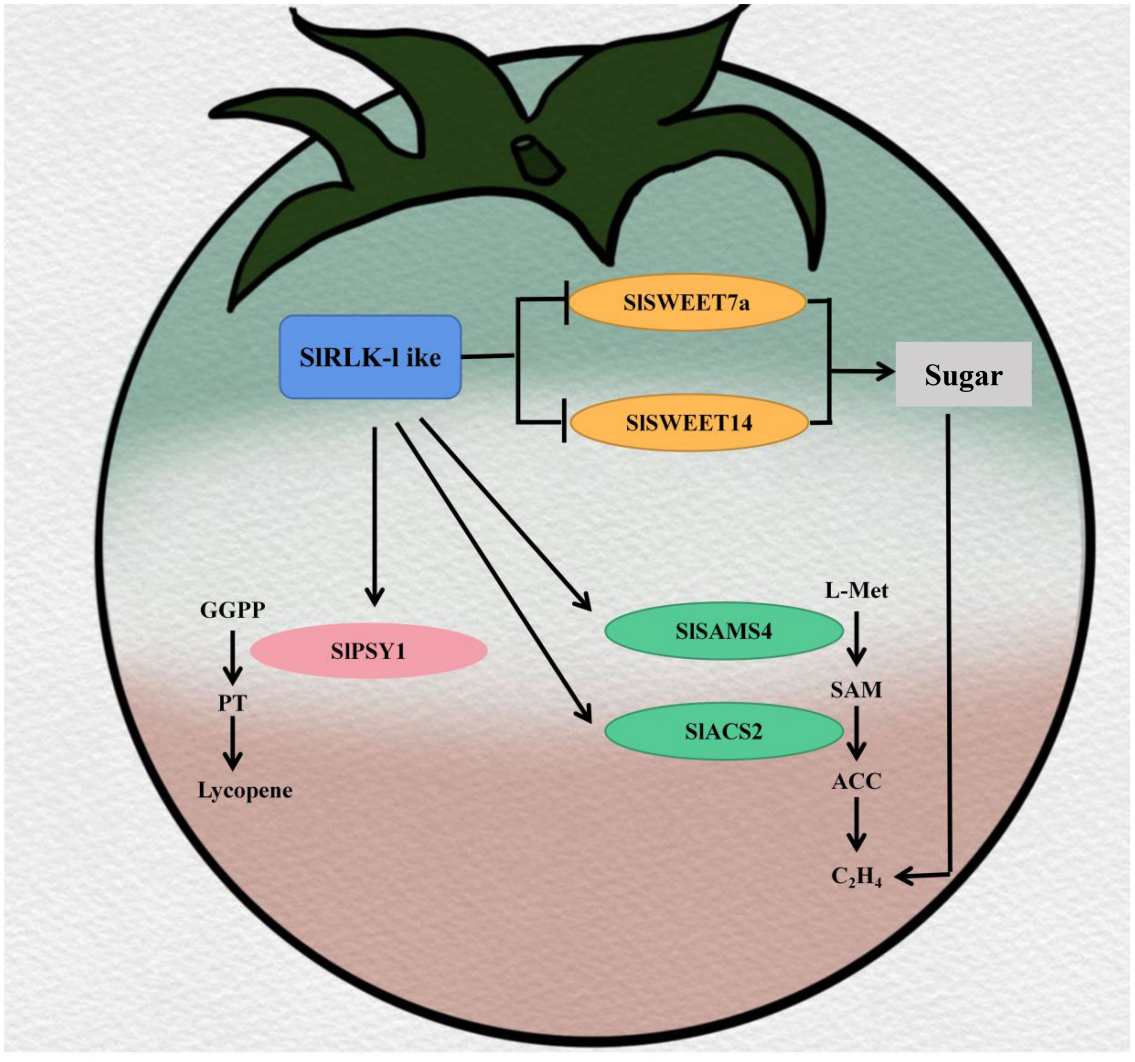
Model of SlRLK-like improving tomato quality in sugar accumulation and the ripening process.

According to the cumulative results, we propose the following model (**Figure 8**). SlRLK-like proteins located on the plasma membrane can interact with SlSWEETs, including SlSWEET7a and SlSWEET14, to improve sugar, in particular Suc, accumulation during tomato fruit ripening. A higher Suc content can then improve ethylene production and accelerate tomato fruit ripening. During the ripening stage, SlRLK-like interacts with SlSAMS4 and SlACS2 to promote ethylene biosynthesis. Furthermore, in the lycopene biosynthetic pathway, SlRLK-like interacts with SlPSY1 to increase lycopene accumulation. Ultimately, SlRLK-like improves tomato fruit quality in myriad aspects, including sugar accumulation, lycopene content, and ethylene production. SlRLK-like, as a receptor-like protein kinase, lacks the kinase domain. However, it participates in regulating the fruit ripening process and promotes the accumulation of sugar in tomato fruits. These findings suggest a brand-new regulatory mechanism for promoting quality and the ripening process in tomato fruits.

## Data Availability

The original contributions presented in the study are included in the article/**Supplementary Material**. Further inquiries can be directed to the corresponding authors.

## Supplementary material

Supplementary Table 1 Primers in the study.

Supplementary Figure 1 Phylogenetic tree of tomato and *Arabidopsis* M/MLD-RLKs.

Supplementary Figure 2 Phylogenetic tree of the SlSWEETs.

Supplementary Figure 3 Fruit weight of *SlRLK-like* transgenic and control fruits.

Supplementary Figure 4 Negative control of bimolecular fluorescence complementation assays in this study.
