# Supplementary figures and images for: Receptor-like kinase SlRLK-like positively regulates sugar accumulation and fruit ripening in tomato
Source: Front Plant Sci. 2025 Aug 20;16:1649082. doi: 10.3389/fpls.2025.1649082 (PMC12406564; doi:10.3389/fpls.2025.1649082)

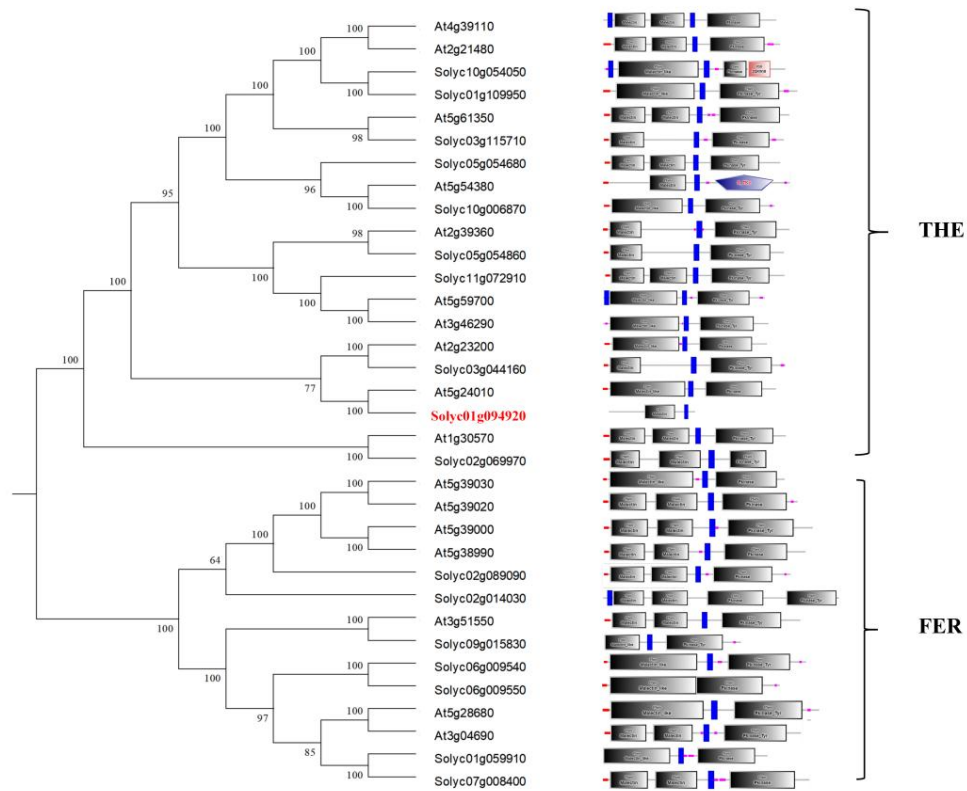

**Fig. S2.** Phylogenetic tree of members of M/MLD-RLKs in tomato and *Arabidopsis*.

Supplement: Supplementary Table 1 — Primers in the study. [file DataSheet1.pdf]

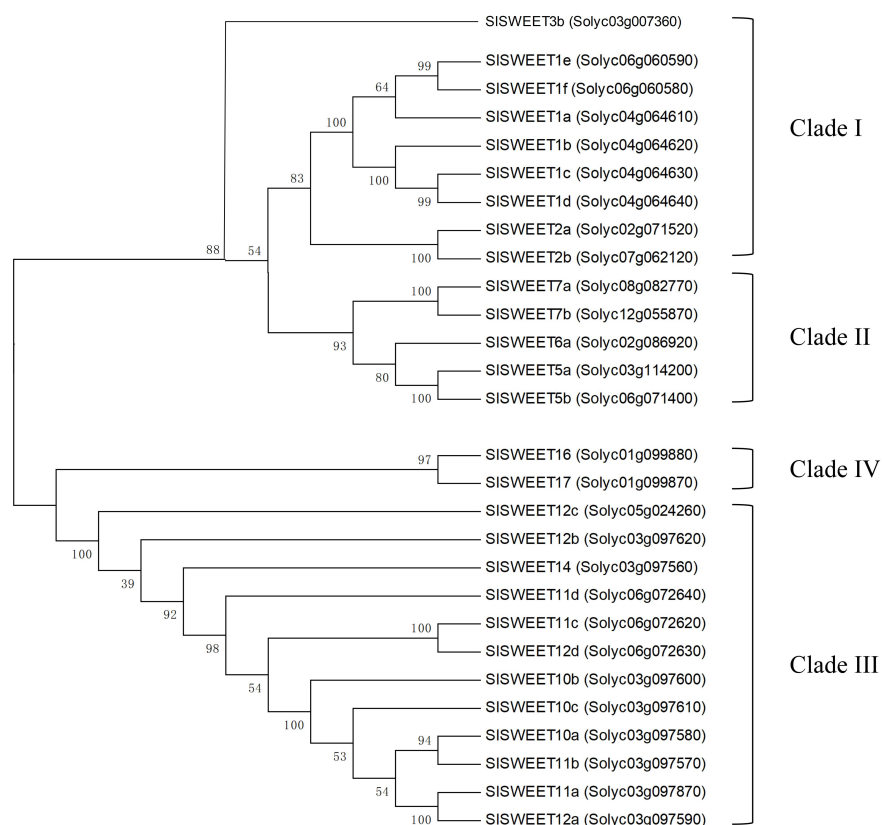

**Fig. S3.** Phylogenetic tree of members of SISWEETs.

Supplement: Supplementary Figure 1 — Phylogenetic tree of tomato and Arabidopsis M/MLD-RLKs. [file DataSheet2.pdf]
