# Supplementary material for: Receptor-like kinase SlRLK-like positively regulates sugar accumulation and fruit ripening in tomato
Source: Front Plant Sci. 2025 Aug 20;16:1649082. doi: 10.3389/fpls.2025.1649082 (PMC12406564; doi:10.3389/fpls.2025.1649082)
Supplement: Supplementary Figure 2 — Phylogenetic tree of the SlSWEETs. [file DataSheet3.pdf]

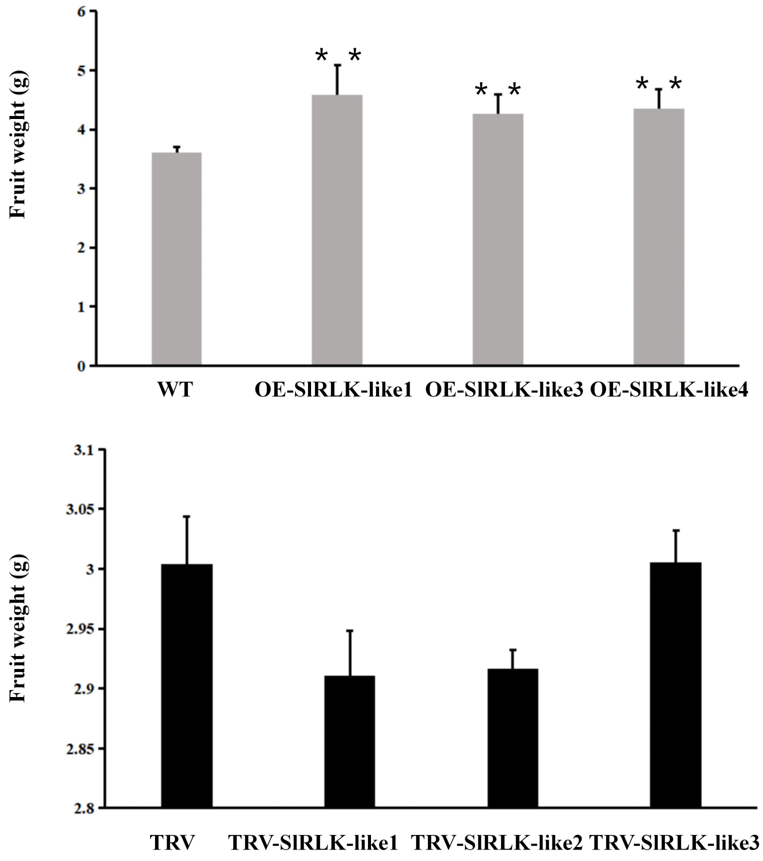

Fig. S4. Fruit weight of *SlRLK-like* transgenic and control fruits. These experiments were performed a minimum of three times. Statistical significance is indicated by *P < 0.05 and **P < 0.01.
