# Supplementary material for: Receptor-like kinase SlRLK-like positively regulates sugar accumulation and fruit ripening in tomato
Source: Front Plant Sci. 2025 Aug 20;16:1649082. doi: 10.3389/fpls.2025.1649082 (PMC12406564; doi:10.3389/fpls.2025.1649082)
Supplement: Supplementary Figure 4 — Negative control of bimolecular fluorescence complementation assays in this study. [file DataSheet5.pdf]

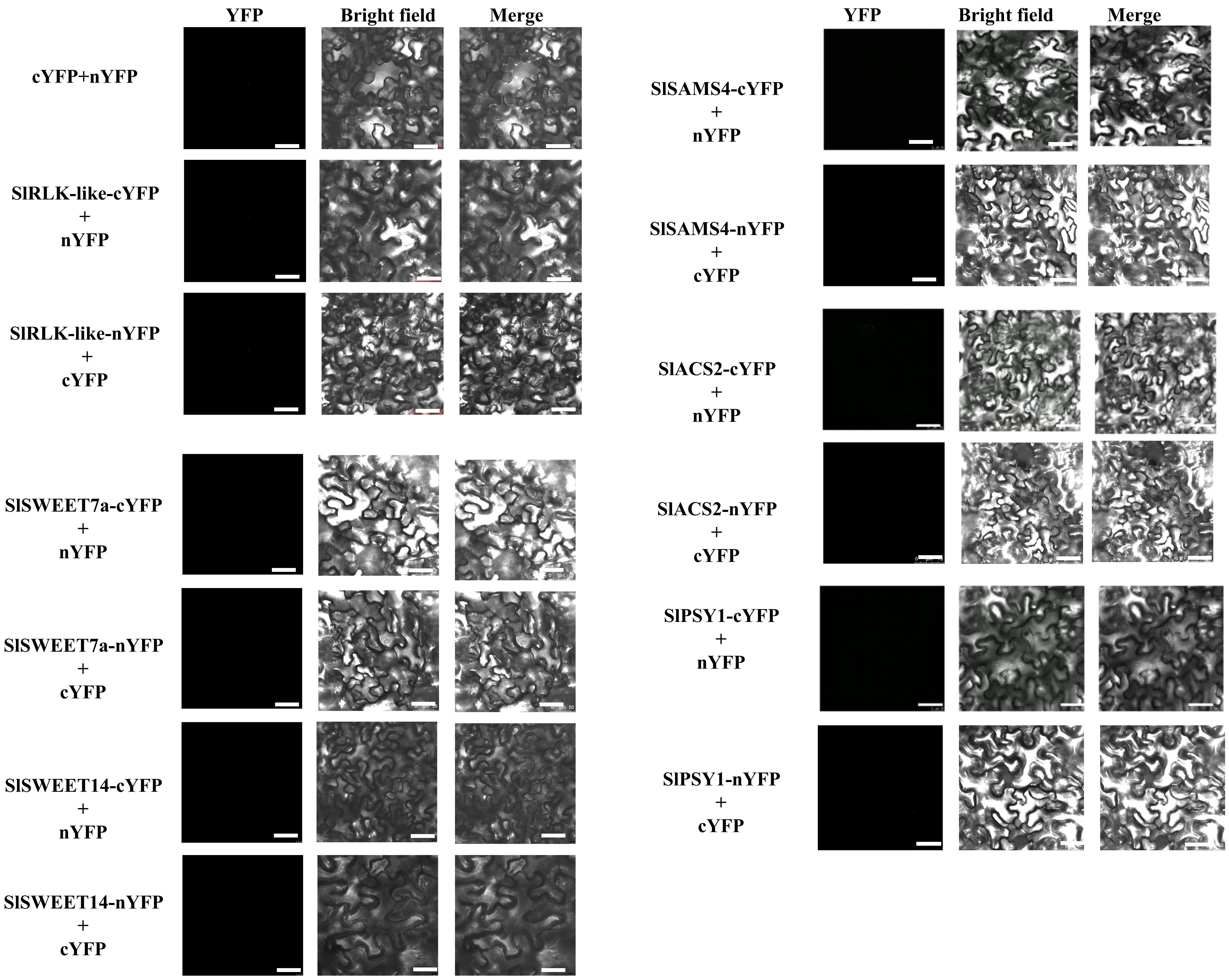

Fig. S1. Negative control of bimolecular fluorescence complementation assays in this study. Scale bars = 25 μm. This experiment was performed a minimum of three times.
